# Giant Polar Displacements via Strain Relaxation in Itinerant Ferromagnet SrRuO_3_ Freestanding Films

**DOI:** 10.1002/advs.202518610

**Published:** 2025-11-23

**Authors:** Xingcan Zhou, Zhangzhang Cui, Qiang Deng, Yechen Wang, Shuyu Dong, Zhou Wang, Qinwen Lu, Jianlin Wang, Qiuping Huang, Zhengping Fu, Bin Xiang, Qingyou Lu, Yalin Lu

**Affiliations:** ^1^ Hefei National Research Centre for Physical Sciences at the Microscale Department of Materials Science and Engineering Anhui Laboratory of Advanced Photon Science and Technology University of Science and Technology of China Hefei Anhui 230026 China; ^2^ High Magnetic Field Laboratory Hefei Institutes of Physical Science Chinese Academy of Sciences Hefei Anhui 230031 China; ^3^ Hefei National Laboratory Hefei Anhui 230088 China

**Keywords:** freestanding oxide film, polar metal, Ru off‐centre displacements, SrRuO_3_, strain relaxation

## Abstract

Polar displacements are conventionally seen as incompatible with metallicity due to charge screening. However, in perovskite oxide heterostructures, cation off‐centring distortions can be achieved through interface engineering strategies—such as ferroelectric proximity and substrate strain—even in centrosymmetric metallic systems. It remains unexplored whether such off‐centring displacements can be stabilized in freestanding oxide films, where substrate‐induced strain is intentionally eliminated. Here, the discovery of substantial Ru off‐centre displacements exceeding 30 pm in freestanding SrRuO_3_ films is reported, achieved by properly controlling the strain relaxation process to engineer an expanded lattice. It is further demonstrated that these Ru off‐centre displacements effectively tune the electronic structure and consequently modify the magnetic properties of SrRuO_3_ thin films. This work shows that tailored strain relaxation strategies can be used to design magnetic polar metals with desired electronic states in otherwise centrosymmetric perovskite oxides.

## Graphical abstract

The discovery of substantial Ru off‐centre displacements exceeding 30 pm in SrRuO_3_ freestanding films is reported, achieved by properly controlling the strain relaxation process to engineer an expanded lattice. The work shows that tailored strain relaxation strategies can be used to design magnetic polar metals with desired electronic states in otherwise centrosymmetric perovskite oxides.

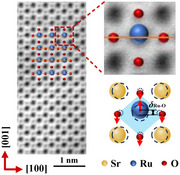

## 1 Introduction

Polar displacements, defined as the off‐centre displacements of ions, are fundamental to many phenomena in condensed matter physics, such as ferroelectricity and multiferroicity.^[^ ^1^, ^2^ ^]^ Such displacements were long thought to be incompatible with metallicity due to charge screening by conduction electrons. However, the experimental realization of polar displacements in conductive oxides has recently attracted growing interest owing to their intriguing functional properties.^[^ ^3^, ^4^, ^5^, ^6^, ^7^, ^8^, ^9^, ^10^, ^11^ ^]^ Among these materials, SrRuO_3_ is an itinerant ferromagnet that adopts a centrosymmetric ABO_3_‐type perovskite structure.^[^ ^12^ ^]^ SrRuO_3_‐based heterostructures have recently emerged as a versatile platform for inducing off‐centring of B‐site cations, making this system one of the rare examples of a magnetic polar metal.^[^ ^13^, ^14^ ^]^ For instance, through proximity to ferroelectrics such as BaTiO_3_, polar displacements can be induced near the interface of SrRuO_3_.^[^ ^14^ ^]^ Furthermore, this ferroelectric proximity‐driven Ru off‐centring breaks the inversion symmetry, leading to an interfacial Dzyaloshinskii–Moriya interaction. It promotes the formation of magnetic skyrmions and gives rise to a topological Hall effect in SrRuO_3_ heterostructures.^[^ ^15^, ^16^, ^17^ ^]^ In addition to the ferroelectric proximity, shear strain from the substrate has also been shown to effectively induce Ru off‐centring and electric polarization in SrRuO_3_ heterostructures.^[^ ^13^ ^]^ Generally, previous studies on the emergence of Ru off‐centring in SrRuO_3_ thin films have primarily focused on interface engineering strategies, utilizing either ferroelectric proximity or substrate strain. In contrast, non‐interfacial approaches to achieving cation off‐centring in SrRuO_3_ and other metallic perovskite oxides remain scarcely explored.

Recently, the advent of fabrication techniques of freestanding oxide films has opened new frontiers in flexible electronics^[^ ^18^, ^19^, ^20^ ^]^ and provides unprecedented opportunities for investigating emergent phenomena in strongly correlated oxide systems.^[^ ^21^, ^22^, ^23^, ^24^ ^]^ By decoupling epitaxial films from their growth substrates through exfoliation, the strain between the films and substrates is eliminated. The strain relaxation involved in fabricating freestanding oxide films is a non‐thermal equilibrium process. Strongly correlated oxides exhibit a complex interplay among spin, orbital, charge, and lattice degrees of freedom, resulting in competing electronic phases with minimal energy separation.^[^ ^25^, ^26^, ^27^ ^]^ A key question is how the strain relaxation process of freestanding oxide films influences their structures and physical properties.^[^ ^28^ ^]^ As demonstrated, many freestanding oxide films exhibit significant differences in both lattice structures and magnetic properties compared to their epitaxial and bulk counterparts.^[^ ^29^, ^30^, ^31^ ^]^ Though some perovskite oxide heterostructures have been shown to develop polar displacements through substrate‐induced strain,^[^ ^7^, ^13^ ^]^ it is conventionally expected that once the films are fully strain‐relaxed from the substrates, they should revert to a centrosymmetric lattice structure, akin to their bulk form. In contrary, it has been reported that strain relaxation can actually promote B‐site cation off‐centring in ultra‐thin freestanding BiFeO_3_ films, a prototypical ferroelectric material.^[^ ^24^ ^]^ This suggests a complex and non‐trivial interplay between strain relaxation and structural evolution in freestanding oxide films.

In this work, we report the discovery of giant Ru off‐centre displacements exceeding 30 pm in SrRuO_3_ freestanding films, where the substrate‐induced strain is intentionally removed. The magnitude of these displacements can be tuned by engineering the strain relaxation process as a function of film thickness. Ru off‐centring is most pronounced in thin freestanding films, whereas it becomes undetectable in thicker samples. We demonstrate that the magnitude of the Ru off‐centre displacements is positively correlated with the expansion of the lattice volume. Furthermore, these substantial displacements significantly modify the electronic structure of the material, thereby altering the magnetic properties of SrRuO_3_ freestanding films. Our findings establish an alternative strategy for creating polar metals and controlling magnetic properties in perovskite oxide thin films.

## 2 Results and Discussion

### 2.1 Fabrication and Structural Characterization of SrRuO_3_ Freestanding Films

**Figure 1:**
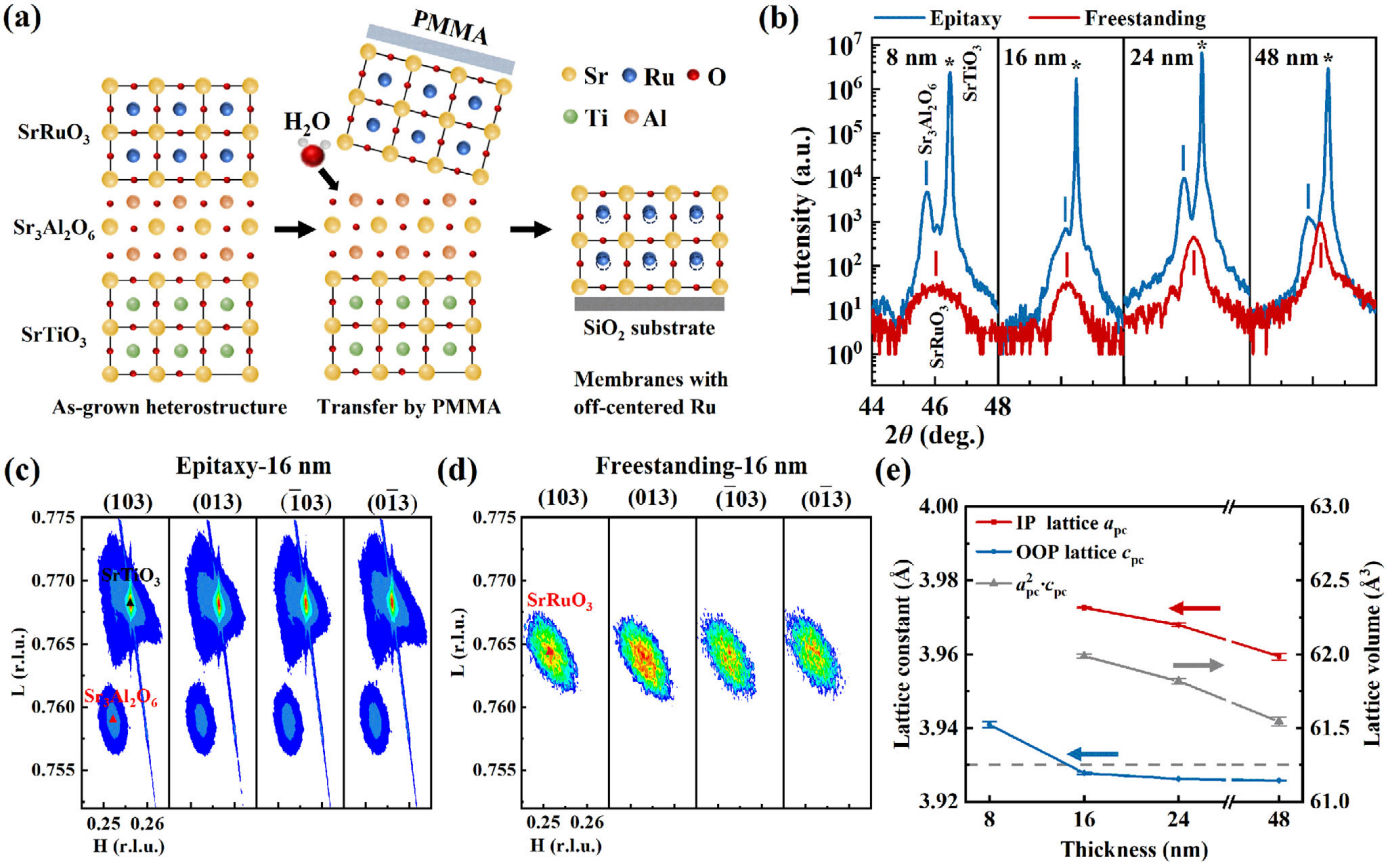
Fabrication and structural characterization of SrRuO_3_ freestanding films. a) Schematic illustration of the fabrication process for SrRuO_3_ freestanding films, highlighting the off‐centring of Ru atoms. b) X‐ray diffraction patterns of SrRuO_3_/Sr_3_Al_2_O_6_ epitaxial films and corresponding SrRuO_3_ freestanding films with thicknesses of 8, 16, 24, and 48 nm. c,d) Reciprocal space maps of a 16 nm SrRuO_3_/Sr_3_Al_2_O_6_ epitaxial film (c) and a SrRuO_3_ freestanding film of the same thickness (d). Conventional RSM measurements could not be performed for the 8 nm freestanding film due to its extremely weak XRD signal. e) Thickness‐dependent in‐plane (IP) and out‐of‐plane (OOP) lattice constants and lattice volume (defined as $a_{\mathrm{pc}}^{2}\cdot c_{\mathrm{pc}}$) in SrRuO_3_ freestanding films. The dashed line indicates the lattice constant of bulk SrRuO_3_. For each thickness, the epitaxial and freestanding films were prepared from the same piece of the sample.

Following the procedures illustrated in **Figure** **1**a (see details in Figure S1a, Supporting Information), we fabricated SrRuO_3_ freestanding films with controlled thicknesses of 8, 16, 24, and 48 nm. The film thicknesses were verified by X‐ray reflectivity (XRR) measurements (see Figure S2, Supporting Information). Throughout the fabrication process, the sacrificial Sr_3_Al_2_O_6_ layers were maintained at a thickness of ≈30 nm. During transfer, poly(methyl methacrylate) (PMMA) was used as a mechanical support to ensure structural integrity, enabling the successful transfer of the SrRuO_3_ freestanding films onto SiO_2_ substrates. The resulting films (see Figure S1b, Supporting Information) exhibit excellent structural continuity under the optical microscope (see Figure S1c, Supporting Information), with no visible cracks or defects.

X‐ray diffraction (XRD) characterization revealed well‐defined diffraction peaks for all four specimens (ranging from 8 to 48 nm) in both epitaxial and freestanding states (see Figure 1b). Given the larger pseudo‐cubic lattice constant of Sr_3_Al_2_O_6_ (close to 4 Å) compared to that of bulk SrRuO_3_ (≈3.93 Å),^[^ ^18^, ^32^ ^]^ this lattice mismatch imposes an in‐plane tensile strain on the SrRuO_3_ films grown on Sr_3_Al_2_O_6_ buffer layers, leading to a corresponding contraction of the out‐of‐plane lattice parameters of SrRuO_3_. This structural adjustment leads to a shift of the SrRuO_3_ diffraction peaks to higher angles, which overlap with the substrate peaks from SrTiO_3_, as shown in Figure 1b. Upon exfoliation from the SrTiO_3_ substrates, the diffraction peaks of the SrRuO_3_ freestanding films become distinctly visible. To further elucidate the structural evolution after exfoliation, reciprocal space mapping (RSM) was performed on both SrRuO_3_/Sr_3_Al_2_O_6_ epitaxial films and SrRuO_3_ freestanding films (see Figure 1c,d; Figure S3, Supporting Information). Both types of films exhibit tetragonal lattice symmetry.

By fitting the diffraction peaks of RSM and out‐of‐plane XRD, we determined the thickness‐dependent in‐plane and out‐of‐plane lattice parameters (denoted in pseudo‐cubic notation as *a*_pc_ and *c*_pc_, respectively) of SrRuO_3_ freestanding films (see Figure 1e). Both *a*_pc_ and *c*_pc_ gradually decrease with increasing film thickness. The out‐of‐plane lattice constant *c*_pc_ varies between 3.92 and 3.94 Å, consistent with the reported value for bulk SrRuO_3_.^[^ ^32^ ^]^ In contrast, the in‐plane lattice constant *a*_pc_ ranges from 3.96 to 3.98 Å, significantly larger than that of bulk SrRuO_3_. This discrepancy results in an unusually expanded lattice volume (defined as $a_{\mathrm{pc}}^{2}\cdot c_{\mathrm{pc}}$) in the freestanding films, which exhibits a pronounced decrease with increasing thickness. It is intriguing that the freestanding films have a larger unit cell volume than that of the bulk, especially in thinner samples. Since strain relaxation is expected once the film is detached from the substrate. This may result from the subtle structural variations across SrRuO_3_ freestanding films of different thicknesses. As strongly correlated oxides typically show competing electronic phases and strain relaxation is a non‐thermal equilibrium process, many freestanding oxide films exhibit distinct lattice structures compared to the bulk, such as LaCoO_3_,^[^ ^29^ ^]^ LaMnO_3_,^[^ ^30^ ^]^ and La_2/3_Mn_1/3_O_3_.^[^ ^31^ ^]^

### 2.2 Atomic‐Resolution Microscopy of SrRuO_3_ Thin Films

**Figure 2:**
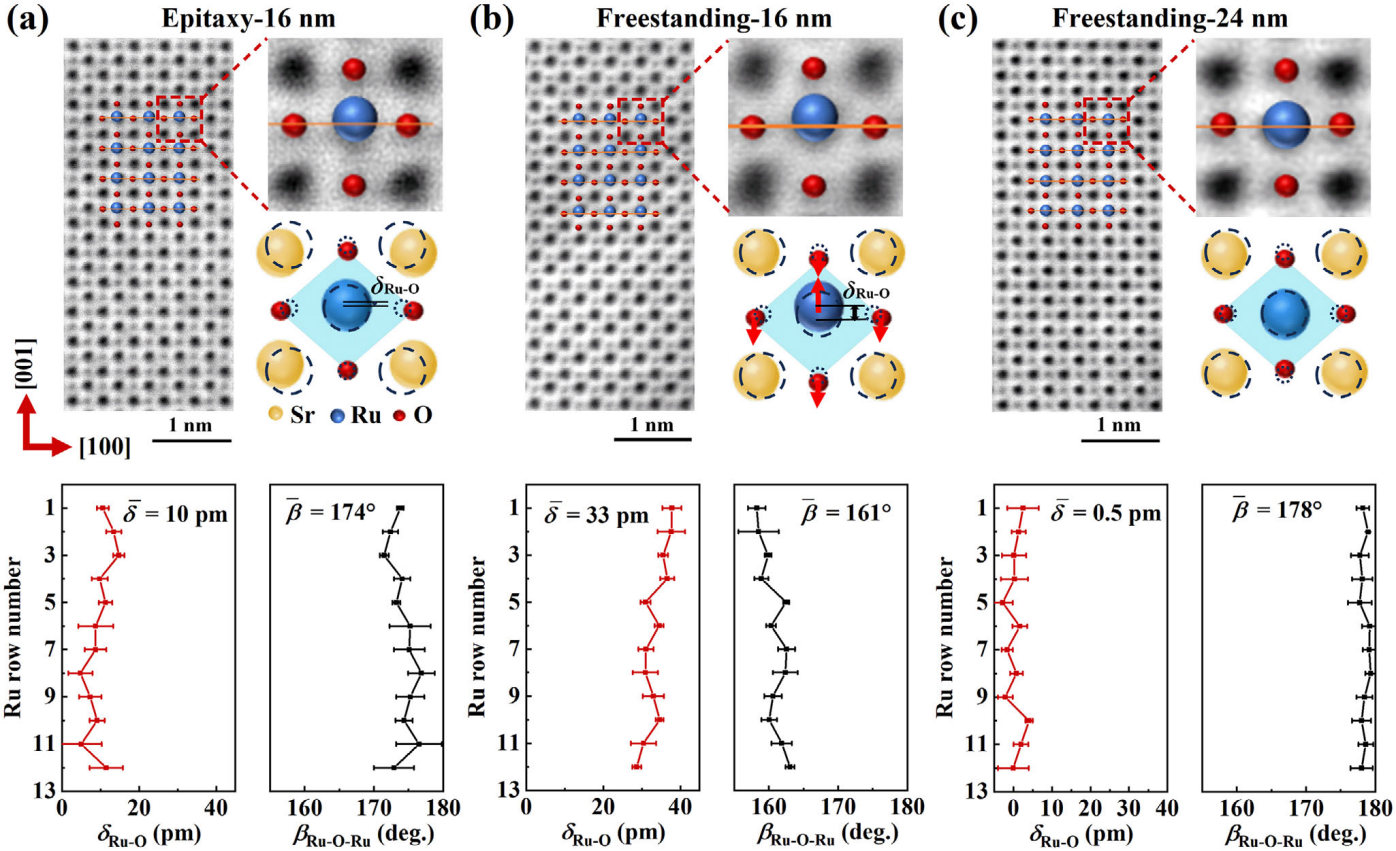
Atomic‐resolution microscopy of SrRuO_3_ thin films. a–c) STEM characterization of 16 nm SrRuO_3_/Sr_3_Al_2_O_6_ epitaxial film (a), 16 nm SrRuO_3_ freestanding film (b), and 24 nm SrRuO_3_ freestanding film (c). The top panels show ABF‐STEM images, and the bottom panels display the corresponding calculated Ru ion displacement magnitudes and Ru─O─Ru bond angles for each atomic layer. In the schematic diagrams, dashed circles indicate the atomic positions expected for a fully strain‐relaxed (ideal) SrRuO_3_ lattice, highlighting deviations in lattice constants and Ru ion displacements from bulk SrRuO_3_. The 16 nm epitaxial and freestanding SrRuO_3_ films were prepared from the same piece of the sample.

To directly visualize the effects of strain relaxation on the atomic‐scale lattice structure of SrRuO_3_ freestanding films, we conducted scanning transmission electron microscopy (STEM) measurements. **Figure** **2** presents annular bright‐field (ABF)‐STEM images of the 16 nm SrRuO_3_/Sr_3_Al_2_O_6_ epitaxial film, along with 16 and 24 nm SrRuO_3_ freestanding films. The Sr, Ru, and O atomic columns are clearly resolved, and their positions were determined via 2D Gaussian fitting (see Figure S4, Supporting Information). Concurrently, the atomic‐scale in‐plane and out‐of‐plane lattice constants were derived from Sr–Sr distances along the [100] and [001] directions, respectively (see Figure S5a–c, Supporting Information). The 16 nm epitaxial SrRuO_3_ film exhibits an average in‐plane and out‐of‐plane lattice constants of 3.992 and 3.873 Å, respectively. After exfoliation, these values have changed to 3.976 Å (in‐plane) and 3.929 Å (out‐of‐plane) in the 16 nm freestanding SrRuO_3_ film. This result provides direct experimental evidence of strain release in the freestanding films.

In the 16 nm SrRuO_3_/Sr_3_Al_2_O_6_ epitaxial film (see Figure 2a), small off‐centre displacements of Ru ions along the [001] axis (denoted as *δ*_Ru‐O_, representing the relative displacement between Ru and O along [001]) are observed. The maximum *δ*_Ru‐O_ in this film is less than 13 pm, with an average value of 10 pm, as statistically derived from the full ABF‐STEM image. These displacements lead to significant deviations of the Ru─O─Ru bond angle along the in‐plane [100] direction (*β*_Ru‐O‐Ru_) from the ideal 180°, yielding an average *β*_Ru‐O‐Ru_ of 174°. The presence of Ru off‐centring suggests the emergence of electric polarization, which is unusual given that SrRuO_3_ is a metal where strong carrier screening typically suppresses polar distortions. Accordingly, SrRuO_3_ films grown directly on SrTiO_3_ substrates usually exhibit centrosymmetric structures.^[^ ^33^ ^]^ However, when subject to epitaxial strain from the substrate, significant Ru off‐centre displacements can occur,^[^ ^13^ ^]^ consistent with the large tensile strain imposed by the Sr_3_Al_2_O_6_ buffer layer in our epitaxial films.

Figure 2b shows the ABF‐STEM image of the 16 nm SrRuO_3_ freestanding film that was derived from the same epitaxial precursor. Remarkably, after exfoliation from SrTiO_3_ substrate, the Ru off‐centring becomes markedly enhanced: the maximum *δ*_Ru‐O_ increases to over 37 pm, with an average of 33 pm—more than three times that of the epitaxial film. Besides, there are noticeable displacements of oxygen atoms relative to the Sr lattice (denoted as *δ*_Sr‐O_, explicitly shown in Figure S5, Supporting Information), which is a natural phenomenon in polar structures.^[^ ^5^, ^34^ ^]^ Concurrently, the average *β*_Ru‐O‐Ru_ decreases to 161°. Such a large B‐site cation off‐centre displacement is exceptional, exceeding values reported for most SrRuO_3_ heterostructures^[^ ^13^, ^14^, ^17^ ^]^ and rivaling that of prototypical ferroelectric BaTiO_3_.^[^ ^35^ ^]^ Intriguingly, Ru off‐centre displacement is not universal across all freestanding films. In the 24 nm SrRuO_3_ freestanding film (see Figure 2c), the Ru off‐centring becomes virtually undetectable, with an average *δ*_Ru‐O_ of only 0.5 pm. The average displacement in the 16 nm film is sixty times larger than that in the 24 nm film. The thicker film also exhibits nearly straight Ru─O─Ru bonds. It should be noted that the Ru off‐centre displacements are consistent throughout the film thickness in both the 16 and 24 nm freestanding SrRuO_3_ films (see Figure S6, Supporting Information). It suggests that the Ru off‐centre displacements are not a function of position along the film thickness direction, but are intrinsically linked to the total film thickness.

Contrary to the conventional view that substrate‐induced strain is essential for triggering off‐centring, our findings reveal that strain relaxation can enhance this distortion. The derived atomic‐scale in‐plane and out‐of‐plane lattice constants (see Figure S5a–c, Supporting Information) directly confirm the distinct non‐equilibrium strain relaxations of freestanding SrRuO_3_ films dependent on the film thickness. The computed average lattice volumes are substantially larger than those of bulk SrRuO_3_, and we find that the magnitude of Ru off‐centre displacements is positively correlated with the unit cell volume of SrRuO_3_ (see Figure S5d, Supporting Information). Notably, the observed enhancement of polar displacements via strain relaxation in thinner SrRuO_3_ freestanding films and their decrease with film thickness are consistent with behaviors reported in ferroelectric BiFeO_3_ freestanding films,^[^ ^24^ ^]^ suggesting that strain relaxation may represent a universal strategy for tuning polar distortions in freestanding oxide films.

### 2.3 SHG Polarimetry and Magnetic Characterization

**Figure 3:**
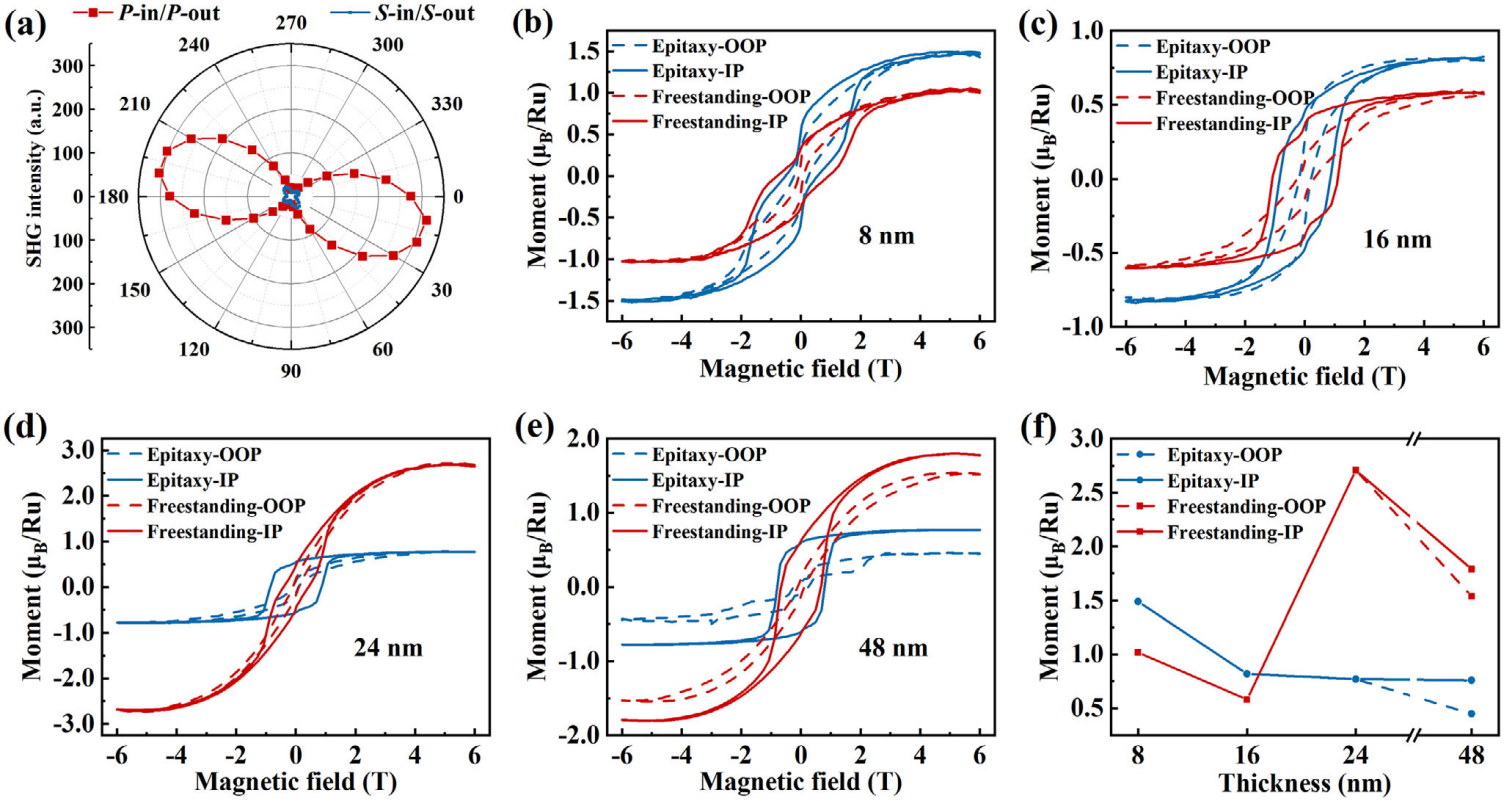
SHG polarimetry and magnetic characterization. a) SHG intensity as a function of polarizer rotation angle *φ* under *P*‐in*/P*‐out and *S*‐in/*S*‐out configurations for the 16 nm SrRuO_3_ freestanding film. b–e) In‐plane and out‐of‐plane magnetic hysteresis loops of SrRuO_3_/Sr_3_Al_2_O_6_ epitaxial films and SrRuO_3_ freestanding films with thicknesses of 8 nm (b), 16 nm (c), 24 nm (d), and 48 nm (e). Measurements were performed at 5 K, with the linear diamagnetic background from the SrTiO_3_ substrates subtracted. f) Thickness dependence of saturation magnetization for both epitaxial and freestanding SrRuO_3_ films. For each thickness, the epitaxial and freestanding films were prepared from the same piece of sample.

The STEM imaging captures the atomic‐scale polar displacements, to characterize the global polar displacements in the 16 nm SrRuO_3_ freestanding film, we performed optical second harmonic generation (SHG) measurements. **Figure** **3**a shows the SHG intensities as a function of polarization rotation angle *φ* under *P*‐in*/P*‐out and *S*‐in/*S*‐out configurations. A clear SHG signal is detected in the *P*‐polarized output, confirming the presence of a polar structure with broken inversion symmetry.^[^ ^5^ ^]^ The symmetry of the SHG profiles suggests a net *mm*2 point group with out‐of‐plane polarization,^[^ ^14^ ^]^ consistent with the STEM observations.

It is well established that structural modulations significantly influence the magnetic properties of SrRuO_3_ heterostructures.^[^ ^33^, ^36^, ^37^, ^38^ ^]^ In the present case, strain relaxation induces thickness‐dependent Ru off‐centre displacements and changes in the lattice volume, which in turn lead to distinct magnetic behaviors across freestanding films of different thicknesses. Figure 3b–e shows magnetic hysteresis loops for 8, 16, 24, and 48 nm SrRuO_3_/Sr_3_Al_2_O_6_ epitaxial films and their freestanding counterparts, measured with in‐plane and out‐of‐plane field orientations. All films exhibit ferromagnetic order with in‐plane magnetic anisotropy, consistent with the tensile strain state.^[^ ^39^ ^]^

Figure 3f summarizes the changes in saturation magnetization of the SrRuO_3_ films before and after exfoliation. In the epitaxial films, the saturation magnetic moment gradually decreases with increasing thickness—for instance, the out‐of‐plane moment drops from 1.50 to 0.76 *µ*_B_/Ru. By contrast, the freestanding films show a distinct trend: the 8 and 16 nm films exhibit reduced moments (1.02 and 0.58 *µ*_B_/Ru, respectively) compared to their epitaxial counterparts, whereas the 24 and 48 nm freestanding films show increased moments, reaching 2.71 and 1.79 *µ*_B_/Ru, respectively. Notably, the 24 nm freestanding film demonstrates a particularly high saturation magnetization. Temperature‐dependent magnetic moments are shown in Figure S7a–d (Supporting Information). The Curie temperature (*T*_C_) of the epitaxial films increases from 130 to 162 K with thickness, while each freestanding film exhibits a slight reduction in *T*_C_ by 4–6 K after exfoliation (see Figure S7e, Supporting Information).

### 2.4 DFT Calculations and XAS Measurements

**Figure 4:**
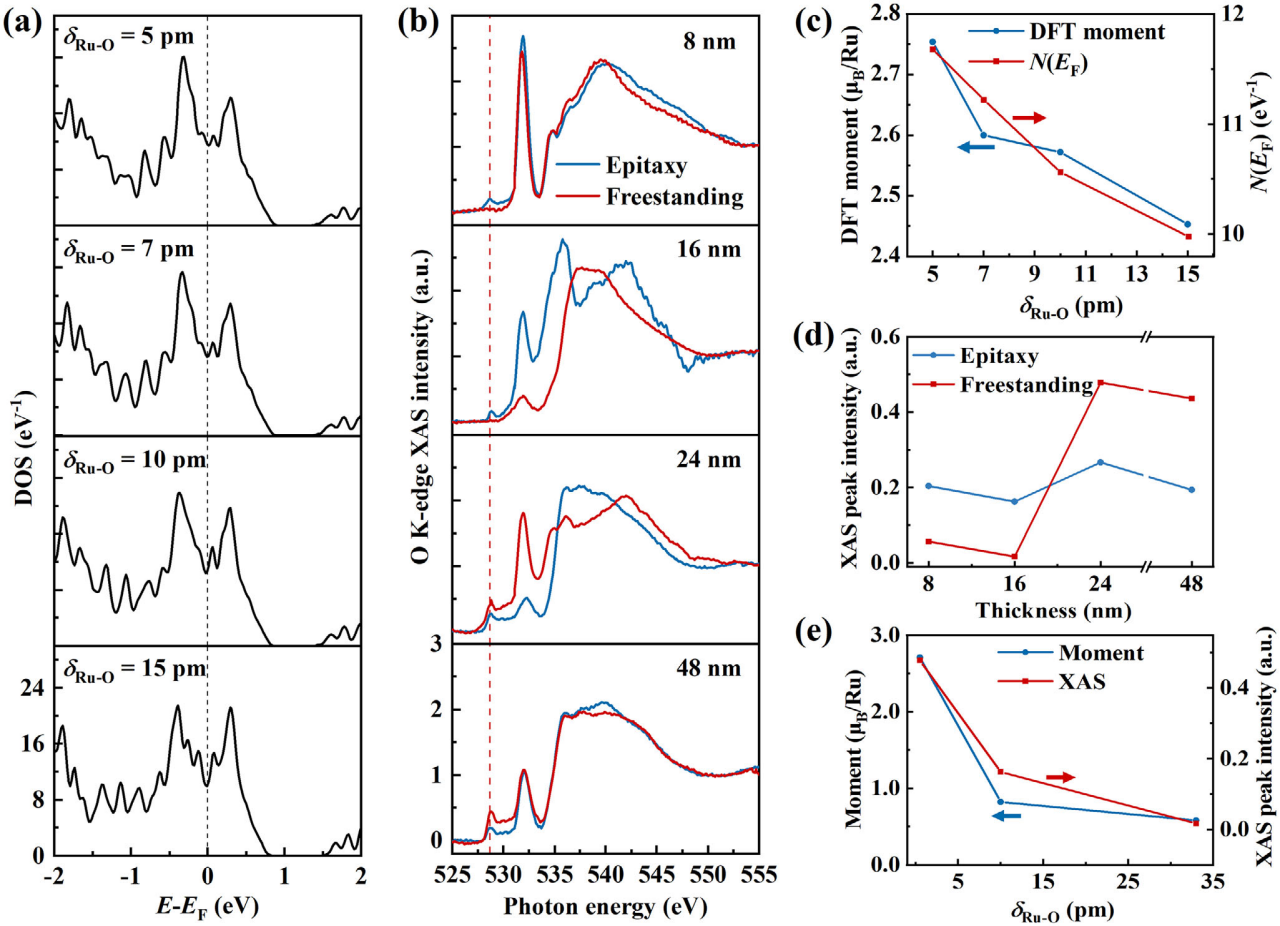
DFT calculations and XAS measurements. a) Density of states (DOS) near the Fermi level in SrRuO_3_ thin films with Ru off‐centre displacement *δ*_Ru‐O_ of 5, 7, 10, and 15 pm. The displacement of oxygen atoms relative to the Sr lattice *δ*_Sr‐O_ is set as half of *δ*_Ru‐O_. b) O K‐edge XAS spectra of SrRuO_3_/Sr_3_Al_2_O_6_ epitaxial films and SrRuO_3_ freestanding films with SrRuO_3_ thicknesses of 8, 16, 24, and 48 nm. For each thickness, the epitaxial and freestanding films were prepared from the same piece of the sample. c) Dependence of calculated saturation magnetic moment and *N*(*E*_F_) on the Ru off‐centre displacement *δ*_Ru‐O_. d) Thickness evolution of the O 2*p*‐Ru 4*d t*_2g_ XAS peak intensity in epitaxial and freestanding SrRuO_3_ films. e) Correlation between the saturated magnetic moment, O 2*p*‐Ru 4*d t*_2g_ peak intensity, and the average Ru off‐centre displacement *δ*_Ru‐O_. Data are shown for the 16 nm SrRuO_3_/Sr_3_Al_2_O_6_ epitaxial film (*δ*_Ru‐O_ = 10 pm), the 16 nm SrRuO_3_ freestanding film (*δ*_Ru‐O_ = 33 pm), and the 24 nm SrRuO_3_ freestanding film (*δ*_Ru‐O_ = 0.5 pm).

Building on the observation that the deviation of in‐plane Ru─O─Ru bond angles from 180° in SrRuO_3_ films originates from the Ru off‐centre displacements along the [001] axis, we conducted density functional theory (DFT) calculations to elucidate the microscopic mechanisms linking Ru off‐centring to magnetic properties. As shown in **Figure** **4**a, the non‐spin‐polarized density of states (DOS) at the Fermi level [*N*(*E*_F_)] increases significantly as the Ru off‐centre displacements *δ*_Ru‐O_ decrease. Figure 4c clearly demonstrates a correlation among the computed saturation magnetic moment, *N*(*E*_F_), and *δ*_Ru‐O_: both the magnetic moment and *N*(*E*_F_) decrease with increasing *δ*_Ru‐O_. According to the Stoner criterion, magnetism is enhanced with higher *N*(*E*_F_),^[^ ^40^ ^]^ which aligns with the calculated trend and previous studies.^[^ ^41^ ^]^

X‐ray absorption spectroscopy (XAS) offers element‐specific insights into electronic structures. Figure 4b displays the O K‐edge XAS spectra for SrRuO_3_/Sr_3_Al_2_O_6_ epitaxial and SrRuO_3_ freestanding films with thicknesses of 8, 16, 24, and 48 nm, measured with the X‐ray beam incident perpendicular to the film plane. Compared to reference SrRuO_3_ spectra,^[^ ^42^ ^]^ the absorption peaks near 529 eV (indicated by a red dashed line) corresponds to transitions from O 1*s* to hybridized O 2*p*‐Ru 4*d t*_2g_ states. Although electric‐dipole selection rules forbid direct O 1*s* to Ru 4*d* transitions, this feature emerges due to strong orbital hybridization, imparting O 2*p* character to the Ru 4*d* states.^[^ ^43^ ^]^ Thus, O K‐edge XAS serves as a sensitive probe of Ru^4+^‐O^2−^ hybridization strength. With the X‐ray beam oriented perpendicular to the film plane, the spectra primarily reflect in‐plane (within the (001) plane) hybridization, making the O 2*p*‐Ru 4*d t*_2g_ peak intensity particularly responsive to Ru off‐centre displacements along the [001] direction.

Figure 4d plots the thickness dependence of the O 2*p*‐Ru 4*d t*_2g_ peak intensity for both epitaxial and freestanding films. The variation in peak intensity closely mirrors the trend in saturation magnetization shown in Figure 3f. Specifically, the 8 and 16 nm freestanding films exhibit reduced intensity compared to their epitaxial counterparts, whereas the 24 and 48 nm freestanding films show an increase. Furthermore, Figure 4e correlates both the saturation magnetic moment and the XAS peak intensity with the average *δ*_Ru‐O_. Both quantities decrease monotonically with increasing *δ*_Ru‐O_. These results demonstrate that the Ru off‐centre displacements—and consequently the magnetic properties—of SrRuO_3_ freestanding films can be systematically controlled through strain relaxation tuned by film thickness. The reduced magnetic moment in thinner SrRuO_3_ freestanding films can be attributed to the significant ion off‐centre displacements observed in these films. First, the DFT calculations suggest that the *δ*_Ru‐O_ reduces the density of states at the Fermi level, which is unfavorable for the ferromagnetic ordering.^[^ ^41^ ^]^ Second, the XAS characterization demonstrates that the *δ*_Ru‐O_ suppresses the O 2*p*‐Ru 4*d* hybridization, which is expected to weaken the magnetic coupling between Ru and O.^[^ ^44^ ^]^

## 3 Conclusion

In summary, we have demonstrated that strain relaxation during the fabrication of SrRuO_3_ freestanding films offers a unique structural strategy to induce Ru off‐centring in SrRuO_3_ thin films. Systematic structural characterizations reveal a thickness‐dependent strain relaxation process in these freestanding films. Atomic‐scale STEM imaging identifies significant Ru off‐centre displacements, with the 16 nm SrRuO_3_ freestanding film exhibiting an average *δ*_Ru‐O_ exceeding 30 pm. The resulting polarity from these displacements is further confirmed by SHG measurements. Magnetic property analysis establishes that the Ru off‐centring modulates the saturation magnetic moments via suppression of O 2*p*‐Ru 4*d t*_2g_ orbital hybridization, as supported by near‐edge XAS results. First‐principles calculations clarify the underlying mechanism by which Ru off‐centring reduces the density of states at the Fermi level. This work illustrates strain‐relaxation control of Ru off‐centring and associated magnetism in SrRuO_3_ freestanding films. Given the established links between Ru off‐centring and emergent phenomena such as the topological Hall effect and polar metallic behavior in SrRuO_3_ thin films, this approach provides a promising platform for exploring novel functional phases arising from the interplay between polar atomic displacements and other electronic orders.

## 4 Experimental Section

### Preparation of SrRuO_3_/Sr_3_Al_2_O_6_ Epitaxial Films

SrRuO_3_ and Sr_3_Al_2_O_6_ were grown on 10 × 10 mm^2^ SrTiO_3_ (001) substrates via pulsed laser deposition (PLD). The Sr_3_Al_2_O_6_ layer was grown at 850 °C under an oxygen pressure of 2 × 10^−3^ Pa using a KrF laser (wavelength 248 nm) with an energy density of ≈1.0 J cm^−2^ and a repetition rate of 2 Hz. The SrRuO_3_ layer was subsequently grown at 720 °C under an oxygen pressure of 13 Pa, using the same laser parameters. After growth, the samples were in situ annealed at 13 Pa oxygen pressure and cooled at a rate of 10 °C min^−1^. Film thickness was controlled via the number of deposition cycles and verified by X‐ray reflectivity (XRR).

### Preparation of SrRuO_3_ Freestanding Films

Poly(methyl methacrylate) (PMMA) was spin‐coated onto the as‐grown heterostructures and baked at 150 °C for 10 min. The sample edges were trimmed to aid water penetration, and the stack was immersed in deionized water. Within ≈15 min, the Sr_3_Al_2_O_6_ layer dissolved, releasing the SrRuO_3_ film, which was then transferred onto a 5 × 5 mm^2^ SiO_2_ substrate. The film‐substrate assembly was dried at 50 °C for 30 min to remove residual moisture. PMMA was removed with toluene, followed by an alcohol rinse to eliminate organic residues, yielding freestanding SrRuO_3_ films.

### XRD Characterization

A Rigaku SmartLab 9 kW X‐ray diffractometer with Cu Kα radiation was used to analyze the crystal structure of the films before and after exfoliation. X‐ray reflectivity (XRR) was employed to determine film thickness, and reciprocal space mapping (RSM) was performed to assess epitaxial quality and in‐plane lattice parameters.

### STEM Imaging

Annular bright‐field (ABF) scanning transmission electron microscopy (STEM) was conducted on a Thermo Scientific Themis Z microscope. Cross‐sectional samples along the pseudo‐cubic (010) plane of SrTiO_3_ were prepared using a Thermo Scientific Helios G4 HX dual‐beam FIB system.

### SHG Measurements

Second harmonic generation (SHG) measurements were performed using a Ti: sapphire femtosecond laser system (*ħω* = 1.55 eV, pulse width = 150 fs, repetition rate = 1 kHz). The beam was focused to a ≈200 µm spot using a 200 mm plano‐convex lens, with an average power of 1 mW to avoid sample damage. Measurements were carried out at 300 K with a 45° incidence angle. Polarization‐dependent SHG signals were collected using a half‐wave plate and linear polarizer in four configurations: *P*‐in/*P*‐out, *P*‐in/*S*‐out, *S*‐in/*P*‐out, and *S*‐in/*S*‐out.

### Magnetic and Electrical Transport Measurements

Magnetic properties were characterized using a Superconducting Quantum Interference Device (SQUID) magnetometer. For temperature‐dependent (*M*–*T*) curves, samples were field‐cooled (1000 Oe) from 300 to 5 K, and magnetization was measured during warming under the same field. Electrical transport properties were evaluated in a Physical Property Measurement System (PPMS).

### DFT Calculations

First‐principles calculations were performed using the Vienna Ab initio Simulation Package (VASP) with the projector‐augmented wave method. A $\sqrt{2}$ × $\sqrt{2}$ × 2 supercell of SrRuO_3_ was built using experimental lattice parameters ($\sqrt{2}$ *a* = $\sqrt{2}$ *b* = 5.63 Å, 2*c* = 7.86 Å). The Perdew–Burke–Ernzerhof (PBE) generalized gradient approximation (GGA) was used, with a Hubbard *U* correction of *U*_Ru_ = 5.5 eV following the previous study.^[^ ^45^ ^]^ A 7 × 7 × 5 Monkhorst–Pack *k*‐point mesh ensured convergence of the total energy, and the electronic self‐consistent iteration was converged to 1 × 10^−6^ eV. The electronic density of states was calculated with a Gaussian smearing of 0.05 eV over a *k*‐mesh of 15 × 15 × 13.

### XAS Measurements

O K‐edge X‐ray absorption spectroscopy (XAS) was conducted at beamline BL12B‐a of the National Synchrotron Radiation Laboratory (NSRL), China. Spectra were acquired at room‐temperature in total electron yield (TEY) mode with the incident beam normal to the sample surface. The beam spot size was ≈1 mm × 0.5 mm.

## Conflict of Interest

The authors declare no conflict of interest.

## Supporting information



Supporting Information

## Data Availability

The data that support the findings of this study are available from the corresponding author upon reasonable request.
