# Supplementary material for: Giant Polar Displacements via Strain Relaxation in Itinerant Ferromagnet SrRuO3 Freestanding Films
Source: Adv Sci (Weinh). 2025 Nov 23;13(8):e18610. doi: 10.1002/advs.202518610 (PMC12884789; doi:10.1002/advs.202518610)
Supplement: Supplementary file 1 — Supporting Information [file ADVS-13-e18610-s001.docx]

Supporting Information

**Giant** **Polar Displacements via Strain Relaxation in Itinerant Ferromagnet SrRuO_3_ Freestanding Films**

*Xingcan Zhou*, *Zhangzhang Cui*,** Qiang Deng*, *Yechen Wang*, *Shuyu Dong*, *Zhou Wang, Qinwen Lu*, *Jianlin Wang*, *Qiuping Huang*, *Zhengping Fu*, *Bin Xiang*, *Qingyou Lu*, *and Yalin Lu**

X. Zhou, Z. Cui, Q. Deng, Y. Wang, S. Dong, J. Wang, Q. Huang, Z. Fu, B. Xiang, Q.-Y. Lu, Y. Lu

Hefei National Research Centre for Physical Sciences at the Microscale, Department of Materials Science and Engineering, Anhui Laboratory of Advanced Photon Science and Technology

University of Science and Technology of China

Hefei, Anhui 230026, China

E-mail: zzcui@ustc.edu.cn; yllu@ustc.edu.cn

Z. Wang, Q.-W. Lu, Q.-Y. Lu

High Magnetic Field Laboratory

Hefei Institutes of Physical Science, Chinese Academy of Sciences

Hefei, Anhui 230031, China

Q. Huang, Z. Fu, B. Xiang, Q.-Y. Lu, Y. Lu

Hefei National Laboratory

Hefei, Anhui 230088, China

**Figure S1.** a) Illustrations of preparation process of SrRuO_3_ freestanding films. Poly(methyl methacrylate) (PMMA) is spin-coated on the surface of SrRuO_3_/Sr_3_Al_2_O_6_ heterostructures to serve as mechanical support during the transfer process. After transferring onto SiO_2_, the PMMA is removed with toluene and ethanol. b) Photograph of a transferred SrRuO_3_ freestanding film. c) Optical microscope images of a SrRuO_3_ freestanding film. d) Scanning electron microscope (SEM) image of a SrRuO_3_ freestanding film. The areas highlighted in red indicate micro holes.

**Figure S2.** a-b) X-ray reflectivity scans of SrRuO_3_/Sr_3_Al_2_O_6_ epitaxial films (a) and SrRuO_3_ freestanding films (b) with thicknesses of 8 nm, 16 nm, 24 nm, and 48 nm.

**Figure S3.** a-b) XRD reciprocal space mapping (RSM) images 24 nm epitaxial (a) and freestanding (b) SrRuO_3_ films. c-d) RSM images of 48 nm epitaxial (c) and freestanding (d) SrRuO_3_ films.


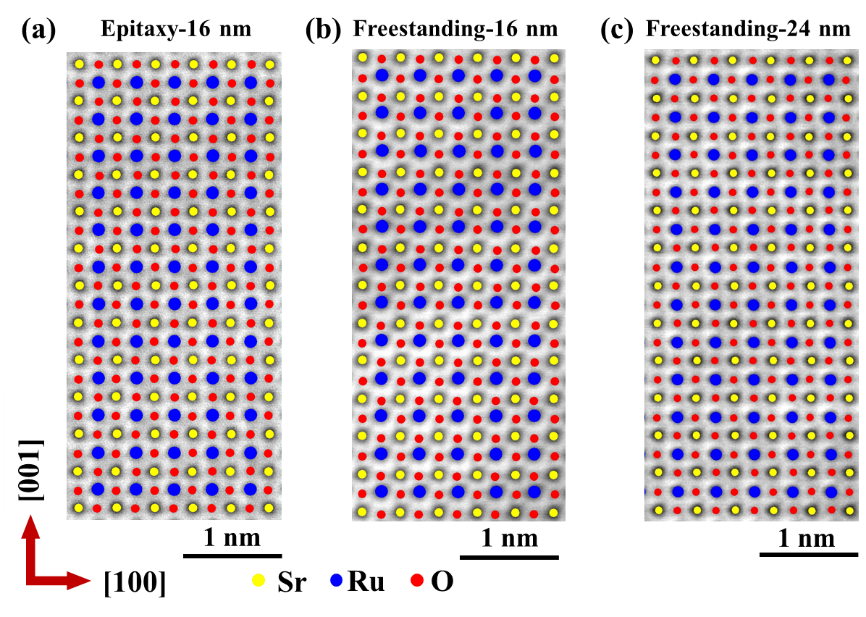


**Figure S4.** a-c) Two-dimensional Gussian fittings of ABF-STEM images of 16 nm SrRuO_3_/Sr_3_Al_2_O_6_ epitaxial film (a), 16 nm SrRuO_3_ freestanding film (b), and 24 nm SrRuO_3_ freestanding film (c).

**Figure S5.** a-c) Fitted in-plane (*a*_Sr-Sr_) and out-of-plane (*c*_Sr-Sr_) lattice parameters, and the average displacements of oxygen atoms relative to the Sr lattice (*δ*_Sr-O_) of 16 nm SrRuO_3_/Sr_3_Al_2_O_6_ epitaxial film (a), 16 nm SrRuO_3_ freestanding film (b), and 24 nm SrRuO_3_ freestanding film (c). d) Correlation between the lattice volume (defined as $a_{\mathrm{pc}}^{2}\cdot c_{\mathrm{pc}}$) and the average Ru off-centre displacement *δ*_Ru-O_.


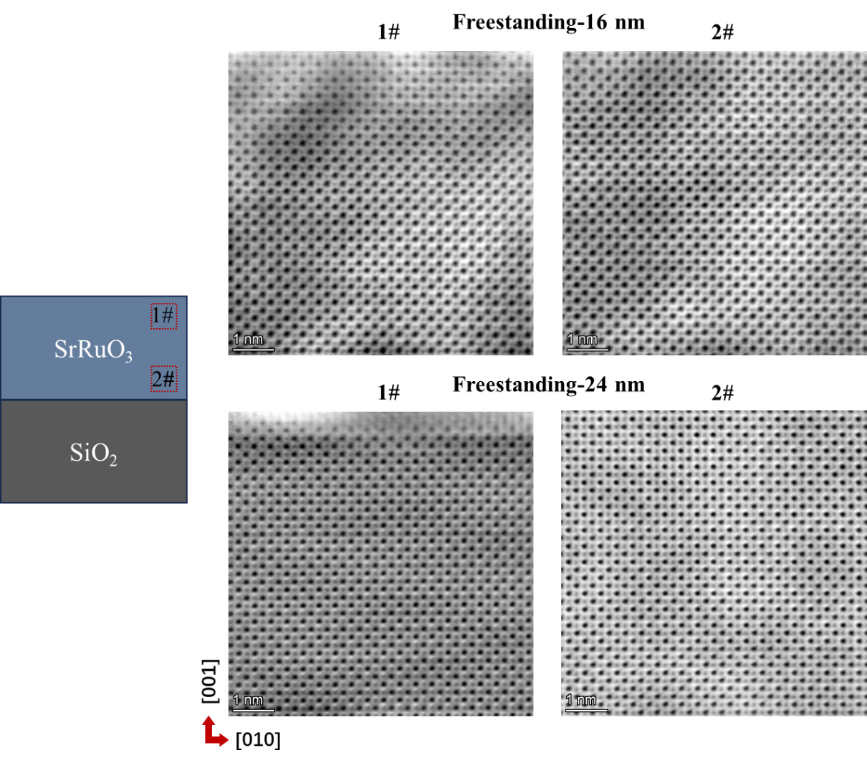


**Figure S6.** ABF-STEM images of the 16 nm and 24 nm freestanding SrRuO_3_ films taken at the top region (1#) and near the interface region (2#).

**Figure S7.** a-b) Temperature-dependent in-plane (IP) (a) and out-of-plane (b) magnetic moments of SrRuO_3_/Sr_3_Al_2_O_6_ epitaxial films with thicknesses of 8 nm, 16 nm, 24 nm, and 48 nm. c-d) Temperature-dependent in-plane (IP) (c) and out-of-plane (d) magnetic moments of SrRuO_3_ freestanding films with thicknesses of 8 nm, 16 nm, 24 nm, and 48 nm. (e) Thickness-dependent Curie tempertures (*T*_C_) of epitaxial and freestanding SrRuO_3_ films.

**Figure S8.** Calculated magnetic moments of SrRuO_3_ without ion off-centre displacements using different *U*_Ru_ values.

**Figure S9.** a-b) Temperature-dependent resistivities of SrRuO_3_/Sr_3_Al_2_O_6_ epitaxial films (a) and SrRuO_3_ freestanding films (b) with thicknesses of 8 nm, 16 nm, 24 nm, and 48 nm. The increased resistivity of freestanding SrRuO_3_ films could be the result of micro holes introduced during the exfoliation.
